# Comparative Analysis of In Vitro Pumps Used in Cardiovascular Investigations: Focus on Flow Generation Principles and Characteristics of Generated Flows

**DOI:** 10.3390/bioengineering11111116

**Published:** 2024-11-05

**Authors:** Noaman Mazhar, Munshi Sajidul Islam, Muhammad Zohaib Raza, SM. Khaled Hossain Mahin, Mohammed Riazul Islam, Muhammad E. H. Chowdhury, Abdulla Al-Ali, Abdelali Agouni, Huseyin C. Yalcin

**Affiliations:** 1 Biomedical Research Center, Qatar University, Doha 2713, Qatar; noaman.mazhar@qu.edu.qa (N.M.); sajidul.islam@qu.edu.qa (M.S.I.); m.zohaibraza@qu.edu.qa (M.Z.R.); 2 Department of Electrical Engineering, Qatar University, Doha 2713, Qatar; sm1912520@qu.edu.qa (S.K.H.M.); ms1513598@qu.edu.qa (M.R.I.); mchowdhury@qu.edu.qa (M.E.H.C.); 3 Computer Science and Engineering Department, Qatar University, Doha 2713, Qatar; abdulla.alali@qu.edu.qa; 4 Department of Pharmaceutical Sciences, College of Pharmacy, QU Health, Qatar University, Doha 2713, Qatar; aagouni@qu.edu.qa; 5 Department of Biomedical Sciences, College of Health Sciences, QU Health, Qatar University, Doha 2713, Qatar; 6 Department of Mechanical and Industrial Engineering, Qatar University, Doha 2713, Qatar

**Keywords:** cardiovascular flow, in vitro pumps, hemodynamics, mechanobiology, shear stress, microfluidics, endothelial cells

## Abstract

A comprehensive analysis of in vitro pumps used in cardiovascular research is provided in this review, with a focus on the characteristics of generated flows and principles of flow generations. The cardiovascular system, vital for nutrient circulation and waste removal, generates complex hemodynamics critical for endothelial cell function. Cardiovascular diseases (CVDs) could be caused by the disturbances in these flows, including aneurysms, atherosclerosis, and heart defects. In vitro systems simulate hemodynamic conditions on cultured cells in the laboratory to study and evaluate these diseases to advance therapies. Pumps used in these systems can be classified into contact and non-contact types. Contact pumps, such as piston and gear pumps, can generate higher flow rates, but they have a higher risk of contamination due to the direct interaction of pump with the fluid. Non-contact pumps, such as peristaltic and lab-on-disk centrifugal pumps, minimize contamination risks, but they are limited to lower flow rates. Advanced pumps including piezoelectric and I-Cor diagonal pumps are focused on improving the accuracy of flow replication and long-term stability. The operational principles, advantages, and some disadvantages of these pump categories are evaluated in this review, while providing insights for optimizing in vitro cardiovascular models and advancing therapeutic strategies against CVDs. The outcomes of the review elaborate the importance of selecting an appropriate pump system, to accurately replicate cardiovascular flow patterns.

## 1. Introduction

The cardiovascular system manages the circulation of nutrients/oxygen throughout the body and the removal of waste/carbon dioxide from the tissues. The heart is the driving pump, vasculature is the distribution network, heart valves ensure unidirectional blood flow, and blood is the delivery fluid. As a result, complex flows with different characteristics are created inside different blood vessels. This system carefully manages and orchestrates complex flow dynamics within different blood vessels, directly influencing the endothelial cells lining the heart chambers and vasculature. Endothelial cells lining the heart chambers and blood vessels are exposed to these complex flows. Hemodynamic forces acting on the endothelial cells are critically important for maintaining a healthy system through biological signals; this is known as mechanobiology. The field of mechanobiology has illuminated the pivotal role of hemodynamic forces acting on these cells in maintaining vascular health. Abnormalities in this balance due to disturbed hemodynamics have been shown to contribute to many cardiovascular diseases (CVDs) such as aneurysms, atherosclerosis, and heart valve and heart chamber defects. CVDs remain the pre-eminent challenge in global health, accounting for nearly one-third of all deaths worldwide in 2019 [1]. In Qatar, as in many regions globally, CVDs are a major health concern.

There are several approaches to investigating CVDs as well as advancements of therapies focusing on hemodynamics. These include clinical observations, in vivo experimentation, and in vitro experimentation. In vitro systems involve culturing cardiac cells within flow chambers and exposing these cells to dynamic flows mimicking hemodynamics in blood vessels. These systems provide a valuable platform to culture cardiac cells and expose them to the native physiological environment for studying the initiation and progression of CVDs as well as for testing pharmaceutical drugs. These systems are also commonly used for tissue-engineering applications, since an important factor for tissue engineered cardiac replacements are mechanical signals by the fluid flow. Such systems are valuable for microfluidics applications such as lab-on-a-chip and organ-on-a-chip as well. In these systems, the replication of pulse flow over cultured cells matching hemodynamic parameters, such as shear stress, is required.

Perhaps the most important component for such a system is the flow generator. In current practice, there are several systems, such as peristaltic pumps, piston pumps, pneumatic pumps, or syringe pumps, used for the generation of continuous fluid flow over cultured cells. However, these pumps often fail to fully replicate the dynamic and complex flow profiles noted in vivo over extended periods while maintaining a sterile environment. This inadequacy represents a significant hurdle in effectively studying CVD progression and creating advanced corrective modalities. This review analyses different types of biomedical pumps used to replicate cardiovascular flow patterns under in vitro settings. By understanding their operational principles, strengths, and limitations, we strive to provide a comprehensive understanding of their suitability in cardiovascular research and therapy development. By evaluating these systems, we will not only obtain guidance for future improvements for in vitro cardiovascular modeling but also contribute to the advancement of therapeutic strategies against CVDs. The upcoming sections of this review will elaborate the specifications of each pump type, checking out their efficacy in replicating physiological flow conditions, and their compatibility with long-term cell culture, also explaining about their role in advancing cardiovascular research and therapeutics.

## 2. Types of Pumps Used for In Vitro Cardiovascular Investigations

In the complex domains of cell and tissue culture, and especially in cardiovascular flow simulation, the selection and application of pumps are of paramount importance. These pumps, varying significantly in their design and functionality, are essential in replicating the dynamic and complex fluidic environment characteristic of the human cardiovascular system. A detailed overview of a wide range of pumps utilized in such applications is elaborated in this paper. Some of the pumps under consideration include peristaltic pumps, syringe pumps, piston pumps, centrifugal pumps, and gear pumps, as well as advanced technologies like I-Cor pumps and piezoelectric pumps. To open the door for a more structured analysis, these pumps are categorized based on key parameters such as their interaction with the flowing fluid, power consumption, and operational complexity within the system. A critical differentiation is made between contact and non-contact pumps. In contact pumps, the fluid directly interacts with the pump, including piston pumps, syringe pumps, vacuum pumps, and gear pumps. Due to this interaction, sterility becomes a challenge, as it is a critical requirement in some cases. These pumps are generally characterized by their ability to generate higher flow rates, but the drawback is the higher risk of contamination. On the other hand, non-contact pumps, such as peristaltic pumps, osmosis-driven microfluidic pumps, and certain centrifugal pump designs, offer a reduced risk of contamination due to their minimal direct interaction with the fluid. This feature is advantageous in sterile environments, despite the fact that it often comes at the expense of lower flow rates. An important example for this category is the lab-on-disk variant, which has a proper balance between maintaining sterility and achieving desired flow characteristics.

These pumps have various applications and are highly dependent on the specific requirements of the experimental setup. In scenarios where pulsatile flow is required, pumps may function independently or in combination with other components, both passive (e.g., dampers and valves) and active (e.g., supplementary pumps). More complex flow patterns mostly make the use of both contact and non-contact pumps, or the employment of multiple pumps of the same type. Therefore, it becomes crucial to select the type of pump appropriately depending on the demands and requirements of the experiment. Recent developments have given rise to hybrid systems that integrate both contact and non-contact pumps. These hybrid systems are designed to compromise between balancing contamination risks and desired flow rates. We will explore three major categories of cardiovascular pumps in the following sections, including non-contact pumps, contact-based pumps, and emerging technologies. Each section will explore the specific features, advantages, and limitations of these pump types, providing a comprehensive understanding of how these diverse mechanisms can be optimally utilized in various research and clinical scenarios.

## 3. Non-Contact Pumps

These pumps operate by keeping the focus on sterility by having a critical separation between fluid and mechanical components, in sensitive experiments. For in vitro cardiovascular experiments and tissue cultures, a sterile environment is very important. Non-contact pumps play an instrumental role in achieving this objective by ensuring aseptic conditions during fluid flow. The design of these pumps avoids direct contact between the fluid and any mechanical components of the pump, such as rotors, rollers, or blades, so it significantly reduces the risk of contamination. Prominent pumps in the category of non-contact pump types are peristaltic pumps, lab-on-disk centrifugal pumps, passive osmosis pumps, and piezoelectric pumps.

### 3.1. Peristaltic Pumps

**Figure 1:**
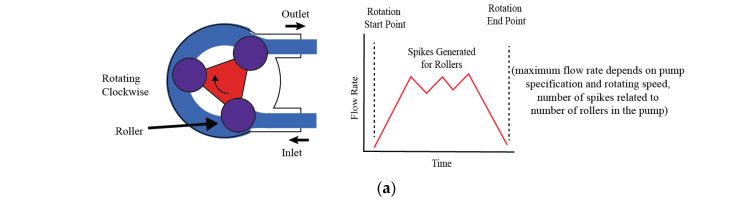

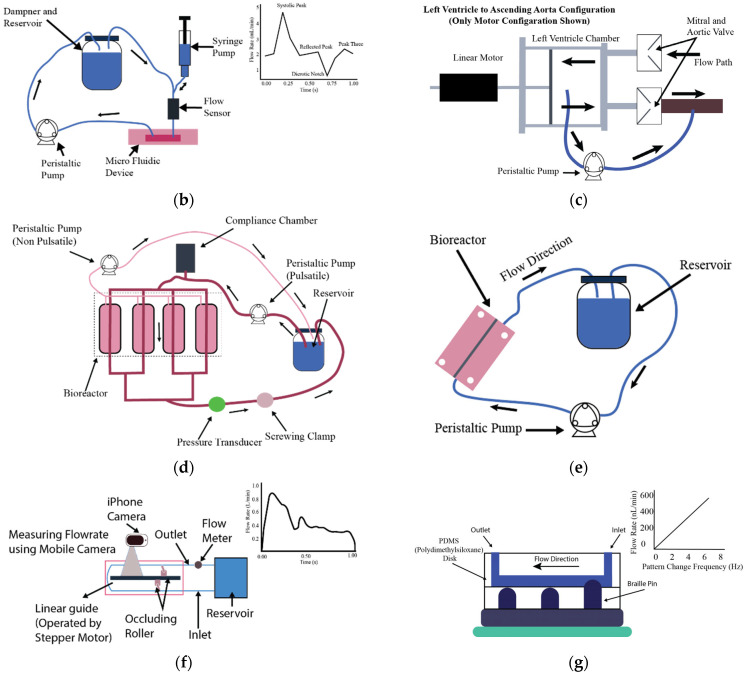
Working principle of peristaltic pump and examples of modifications. (**a**) Generic peristaltic pump construction schematic, (**b**) schematic of a pulsatile flow mimicking setup using a peristaltic pump and one syringe pump [2], (**c**) schematic diagram of the left ventricle chamber using peristaltic pump and piston pump [3], (**d**) pulsatile bioreactor system, (**e**) simple bioreactor setup using peristaltic pump [4], (**f**) schematic of a novel roller pump for physiological flow mimicking setup [7], (**g**) schematic diagram illustrates a Braille display pump [6].

The working principle of peristaltic pumps is as follows: when the rotor rotates, rollers move across the length of the tube in a way that causes compression of the tube, with two of its rollers, and some amount of fluid is held in between these rollers, which is then moved forward with further rotation. The adjustment in these factors can precisely control the flow rate, which makes the peristaltic pump versatile and suitable for a number of applications. The peristaltic pump is a type of positive displacement pump, which is widely recognized in fluidic systems. Positive displacement is an efficient and distinctive mechanism which is characterized by the continuous compression and subsequent relaxation of a flexible tube, which is facilitated by rollers attached to a rotating rotor. When the rotor rotates, these rollers move across the length of the tube, compressing it and thus creating a sealed segment that contains a fixed volume of fluid. All this rotation happens in a sequential and continuous way, resembling the peristalsis motion observed in biological systems, the same as the gastrointestinal tract. The movement of these rollers along the tube creates a wave-like flow [Figure 1a]. This flow can be both pulsatile and linear, due to the nature of the compression and movement of the fluid through the tube. The flow rate through the tube is dependent on a variety of factors, including the speed at which the rotor rotates, the number and size of the rollers, and the diameter of the tube itself. Adjustments in these factors can precisely control the flow rate, which makes the peristaltic pump versatile and suitable for a wide range of applications.

As it is a non-contact pump, the fluid in a peristaltic pump is completely isolated from all the mechanical components of the peristaltic pump, while flowing through a flexible tube. This attribute makes it valuable in applications such as in pharmaceutical production, medical procedures, and other sterile processes. In designs where sterility must be maintained and the prevention of contamination is crucial, this design is very advantageous. The ability to control the flow rate by adjusting the rotor’s speed is another key benefit, allowing for a wide range of applications, from minute fluid transfers to larger volume handling. Additionally, peristaltic pumps have the ability to handle various types of fluids, including viscous fluids and biological materials containing fluids, without damaging or degrading the fluid. This pump has a simple design which is not just limited to its operation, but extends to its maintenance as well. The main component for fluid to flow is the tube, which can easily be replaced without the need for comprehensive disassembly of the pump. Easy maintenance, reliability, and the ability to control fluid handling strengthen the peristaltic pump’s role as a versatile and fundamental tool to be used in different areas, including scientific, industrial, and medical applications. Its unique working principle efficiently harmonizes the mechanical aspects of fluid propulsion with the critical requirements of fluid integrity and contamination control.

A schematic of the working principle of a generic peristaltic pump is shown in Figure 1a. Although peristaltic pumps generate unidirectional, oscillatory, and somewhat pulsatile flows, they commonly lack the ability to accurately replicate physiological cardiovascular flows. Flow inconsistency is another concern. To overcome these limitations, many modifications have been proposed to improve flow characteristics for better mimicking of cardiovascular conditions. Bouhrira et al. [2] enhanced flow regularity by integrating a peristaltic pump with a damper and a syringe pump, as shown in Figure 1b. By adding a damper in series with the peristaltic pump, the authors were able to generate consistent physiologically relevant flow profiles. Similarly, Rezaienia et al. [3] explained the innovative integration of a piston pump and a peristaltic pump into a hybrid system which can produce intricate flow patterns. The schematic diagram illustrating only the left ventricular chamber with one peristaltic and one piston pump in series is provided in Figure 1c. By arranging multiple pumps in both series and parallel configurations, the study successfully mimicked the diverse conditions of the left ventricular chamber. Through this configuration, they achieved an improved pulse wave analysis in their research. Helms et al. [4] demonstrated the flexibility of dual peristaltic pumps within a heart–lung simulation system, emphasizing their applicability for physiological modeling. In the setup, there were two peristaltic pumps: one modified to mimic a heart-lung machine pump providing pulsatile flow, and another non-pulsatile pump for recirculating fluid from the bioreactor. The key component was the modified pulsatile peristaltic pump, which was programmed to replicate the pressure conditions of the heart and lungs, shown in Figure 1d. This study effectively applied a pulsatile and a non-pulsatile peristaltic pump, in series, with a reservoir to generate a pulsatile bioreactor perfusion system. Eoh et al. [5] further highlighted its application in tissue engineering and regenerative medicine applications. They utilized the peristaltic pump with a bioreactor and a reservoir and established a consistent linear flow [Figure 1e]. To improve upon the generation of pulsatile flow, novel peristaltic pump designs have been introduced. These designs incorporate elements such as Braille displays and a combination of stepper and servo motors [Figure 1f]. In Braille display technology, micro actuators constructed from piezoelectric bimorphs are employed to manipulate fluid dynamics within a specialized channel–membrane configuration. These actuators exert force on the fluid based on programmed Braille movements, directing the flow accordingly. The configuration is characterized by a flow rate capability of up to 600 nl/min. While this flow rate is relatively low for a broad range of applications, it proves highly effective for scenarios requiring precise fluid control. The compact size of the pump, inherited from its Braille display origins, allows for the integration of multiple inlets and outlets, facilitating intricate flow management. This feature is particularly advantageous in lab-on-a-chip experiments, where precise fluid control is important. In Figure 1g, the schematic diagram illustrates a Braille display pump developed by Gu, Wei et al. [6], where the Braille display’s movable pins act as programmable pumps and valves, enabling precise control over microfluidic flows. This system supports a wide range of fluidic operations such as rapid mixing, laminar flows, and segmented plug flow within the same channel architecture. The device could seed cells, compartmentalize them, and sustain long-term cultures, specifically of mouse myoblast cells, for up to three weeks under continuous perfusion. The system’s versatility was demonstrated by controlling cellular proliferation and differentiation through different flow rates and channel configurations. This low-cost, portable, and programmable system opens new opportunities for microscale tissue engineering and high-throughput biological studies. Despite its advantages, the complexity of the setup may pose challenges in scaling for broader applications. Chong et al. [7] engineered an advanced peristaltic pump that operates on principles like traditional peristaltic pumps but differs significantly in construction. Rather than employing a DC or stepper motor attached to rollers to compress the flexible tubing and propel the fluid, this design utilizes a linear motor. The linear motor precisely drives two servo motors in linear manner, which are responsible for squeezing the flexible pipe, thereby facilitating the flow of fluid through the conduit. In their study, the authors demonstrated that the flow profile produced by the reciprocating roller pump closely matched the programmed profile, achieving a remarkable similarity index of 0.98. In their study, Balagaddé et al. [8] utilized a peristaltic pump integrated into a micro hemostat to generate continuous flow at a linear velocity of approximately 250 µm/s. This setup allowed for semi-continuous, planktonic bacterial growth, effectively preventing biofilm formation and enabling long-term monitoring of cell populations under controlled flow conditions. Gómez-Sjöberg et al. [9] utilized an on-chip peristaltic pump to deliver precise media flow rates of 6.5 nL/s, with a maximum shear stress of 0.05 N/m^2^. This setup allowed for automated, long-term stem cell culture and differentiation studies, supporting up to 96 chambers with individual addressability. The system provided controlled, pressure-driven flow for media exchange, ensuring minimal cross-contamination and consistent culture conditions over a 7–9-day period.

### 3.2. Osmosis Pumps

**Figure 2:**
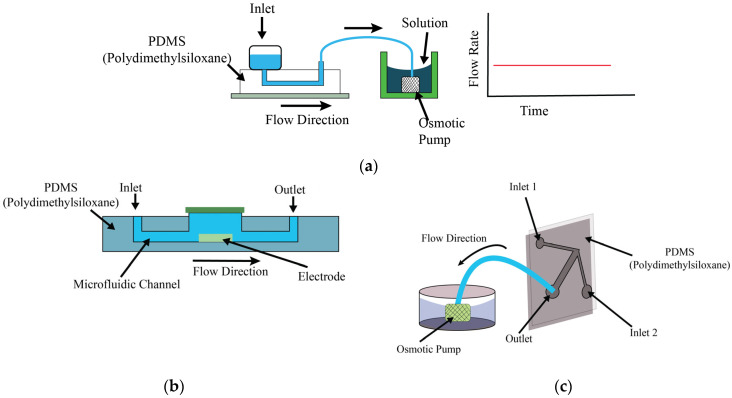
Working principle of osmosis pumps and example modifications. (**a**) Generic osmosis pump construction schematic, (**b**) schematic diagrams of electrofusion chip driven by surface tension [10,11], (**c**) schematic of gradient generation by an osmotic pump setup [12,13].

In microfluidics, osmosis-driven pumps represent a paradigm shift, as these pumps operate autonomically from external sources of electrical power. Osmosis-driven pumps work on the principle of the natural process of osmosis, which is the movement of solvent molecules across a semipermeable membrane from a region of lower solute concentration to a region of higher solute concentration. This is a controlled movement which equalizes solute concentrations on both sides of the membrane by osmotic pressure; therefore, these pumps are recognized as “passive” pumps. In these pumps, the semi-permeable membrane plays an important role, as it allows the solvent molecules, generally water, to pass, while not allowing the solute molecules to pass. When the solvent on one side of the membrane (normally the side with lower solute concentration) comes into contact with the membrane, it spreads through to the other side (where the solute concentration is higher), thus generating fluid flow as shown in Figure 2. The flow rate in an osmosis-driven pump is controlled by the osmotic pressure gradient, which is the difference in solute concentration across the membrane. A higher osmotic pressure results in a larger gradient, which drives the solvent molecules through the membrane more rapidly. Also, the flow rate is affected by the contact area of the membrane because more solvent molecules can pass through at once through a larger surface area. This method of fluid movement results in a unidirectional flow at a rate typically measured in microliters per minute (μL/min). The flow continues if there is an osmotic pressure gradient, which makes these pumps especially useful for applications that require sustained and consistent fluid movement over long periods of time. These pumps are simple and autonomous in terms of the requirement of an external power source, which makes them efficient in various microfluidic applications, mostly when a controlled and contamination-free environment is required. These systems are often configured in an open-loop setup, with designated inlet and outlet points to facilitate continual fluid introduction and maintain a stable flow rate. Although osmosis-driven pumps offer several benefits, one of the challenges is the dependence on the osmotic pressure gradient, which might limit their applicability in scenarios where highly variable flow rates or pressures are required. They are very useful in cell culture studies, pharmaceutical research, and other microbiological applications where maintaining environmental stability is essential. A demonstration, through various experiments, that osmosis-driven passive pumps can serve as reliable systems for sustained flow generation over extended periods is given by Ju [10,11] and Park [12,13]. These osmotic pumps are characterized by their reliance on key factors such as the concentration of the osmotic agent and surface tension. These pumps are able to achieve nanoliter flow rates for prolonged durations in sterile conditions.

### 3.3. Centrifugal Pumps

**Figure 3:**
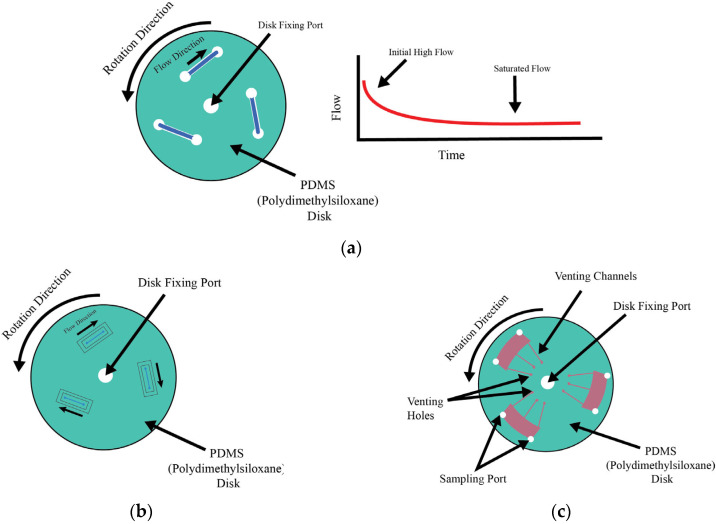
Working principle of non-contact centrifugal pumps and example modifications. (**a**) Generic centrifugal pump or LAB on disk construction schematic, (**b**) schematic of cell positioning inside microfluidic device experiment [14], (**c**) schematic of cell culture system in LAB in disk setup [15].

Centrifugal pumps in microfluidic systems are a critical component in lab-on-a-chip applications. In microfluidic systems, they work on the principle of centrifugal force, which drives the fluid through channels, making this pump type a unique and precise method of controlling fluid flow. The main component of this operation is a rotating disk or rotor, which, when rotating at high speed, exerts an outward force on the fluid within the embedded microfluidic channels. The primary driver for propelling the fluid through the channels is the centrifugal force, determining the flow direction and rate [Figure 3a]. Depending on the design and application, these pumps may or may not be in contact with the fluid. Here, we explain the non-contact application.

The modulation of flow in these systems is a sophisticated process influenced by several relevant factors. Fluid propulsion is controlled by the rotational speed of the disk: when the speed is higher, the centrifugal forces will be greater, leading to faster fluid propulsion. Similarly, lower speeds result in reduced force and slower flow rates, allowing for precise control over the movement of the fluid. The design and dimensions of the channels also play a vital role in the rate of flow. Factors like the width, length, and overall layout of these channels directly affect the flow characteristics. A higher resistance would be faced by the flow when the narrower channels are used, which will reduce the flow rate, while wider channels facilitate easier fluid passage, which helps increase the flow rate. Additionally, the physical properties of the fluid itself, such as viscosity, density, and surface tension, significantly affect its response to the applied centrifugal force. More-viscous fluid flows slower compared to less-viscous fluid under similar conditions of rotational speed and channel design. A high degree of control can be achieved over the flow rates by these same explained factors, from the speed of rotation to the fluid properties and channel design, which can vary from very slow (tens of nanoliters per minute) to relatively fast (hundreds of microliters per minute) flows. This versatility and precision in centrifugal pumps is suitable for a broad spectrum of microfluidic applications, ranging from complex biochemical experiments to innovative lab-on-a-chip systems, which makes them important in the dynamic field of microfluidics. The comparison of characteristics of non-contact pumps, highlighting the flow rate, flow patterns, shear stress, application sectors, and key advantages and disadvantages, is shown in Table 1.

## 4. Contact Pumps

Contact pumps have direct engagement with the fluid, such that the mechanical parts like rotors and rollers come into direct contact with fluid, influencing their use in cell or tissue culture. Centrifugal, syringe, piston, gear, I-Cor, and diaphragm pumps are some common types of contact pumps, chosen according to specific needs. These pumps have some general benefits which include the handling of higher flow rates and versatility in handling various fluid viscosities. Their main drawback is the risk of contamination due to direct contact with the fluid. This necessitates strict sterilization to mitigate risks, especially in sensitive applications like cell culture.

### 4.1. Centrifugal Pumps

**Figure 4:**
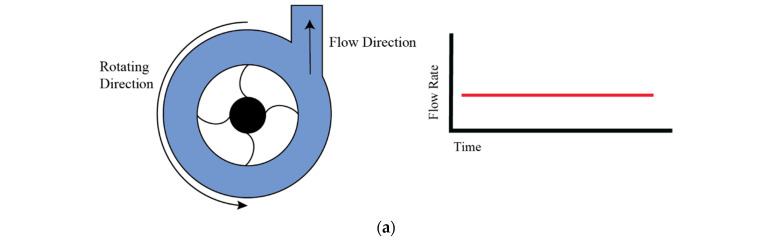

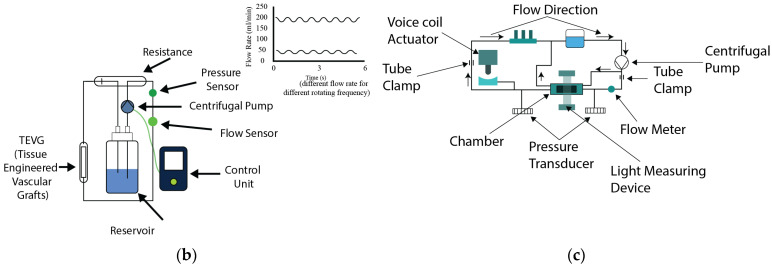
Working principle of centrifugal pump and examples of modifications. (**a**) Generic centrifugal pump or LAB on disk construction schematic, (**b**) schematic of cell positioning inside microfluidic devices experiment [28], (**c**) schematic of cell culture system in LAB in disk setup [29].

In centrifugal pumps, the fluid is moved through the rotation of an impeller or rotor, which imparts kinetic energy to the fluid, causing it to move outward and creating a pressure differential that facilitates a consistent flow of liquid. This principle allows for the production of both constant and variable flow rates, depending on the operational conditions and the pump’s speed, which can be adjusted to achieve desired flow patterns such as pulsatile flows. A schematic representation of a centrifugal pump’s general construction is illustrated in Figure 4a. As presented in the diagram, there is a rotor blade which induces a pressure differential within the pump on rotation. The force behind the movement of liquid is driven by this differential, which efficiently facilitates flow through the pump’s operational pathway. This visual representation explains the fundamental working principle of centrifugal pumps, emphasizing the role of the rotor blade in the generation of fluid movement. According to the design and application, these pumps can be in non-contact or in contact with the fluid. Here, we explain the contact application. Wolf et al. [28] developed a compact, portable, and versatile bioreactor designed specifically to create an optimal environment for the fabrication of tissue-engineered vascular grafts, as detailed in their schematic design presented in Figure 4b. Here, a centrifugal pump is regulated by a custom controller, which is capable of producing a broad spectrum of hydrodynamic conditions according to different pressure requirements. This feature underscores the bioreactor’s adaptability and its potential to closely mimic physiological conditions, highlighting the critical role of controlled fluid dynamics in tissue engineering applications. Diamantouros et al. [29] introduced a bioreactor specifically designed for tissue engineering of vascular grafts, incorporating a pulse-free centrifugal pump to ensure constant flow generation, alongside a linear magnetic actuator instead of using a single centrifugal pump to generate pulsatile flow. This actuator works by applying controlled and programmable pressure pulses, which enables the creation of variable cardiovascular profiles ranging from less than 1 Hz to 5 Hz. The innovative approach helps in the simulation of physiological conditions necessary for vascular tissue engineering. The schematic representation of this bioreactor, including its key components and their functional integration, is depicted in Figure 4c, highlighting the system’s capability to replicate the dynamic environment of the cardiovascular system.

The use of centrifugal pumps is not without its challenges. The necessity for direct contact between the pump’s impeller and the fluid introduces the potential for contamination, which is of particular concern in applications requiring high levels of sterility such as tissue culture or pharmaceutical manufacturing. Intensive sterilization protocols, including UV treatment and chemical cleaning, are essential to reduce these risks. Furthermore, the design of centrifugal pumps inherently supports only forward flow, limiting their suitability in applications where reverse flow control is needed. Due to the mechanical interaction between the impeller and the fluid, there is a risk of mechanical shear, which can damage delicate cells in biological experiments. Even though it has such limitations, its capability to manage the flow of a large volume of fluid, sometime 1 L per minute, makes these pumps very useful in the applications where large volumes of fluid need to be moved. This capacity, combined with their adaptability and ability to handle a variety of flow rates and patterns, positions centrifugal pumps as a preferred choice in many experimental and clinical settings. Balancing their operational advantages against the potential challenges is a critical consideration in selecting centrifugal pumps for specific applications.

### 4.2. Piston Pumps

**Figure 5:**
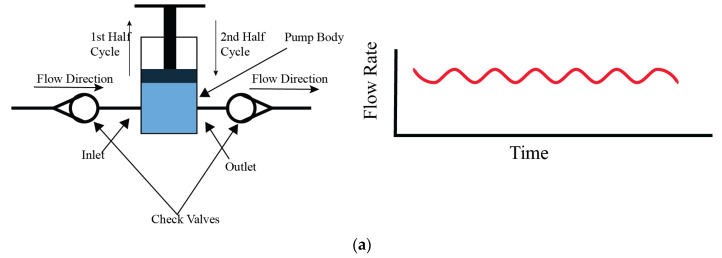

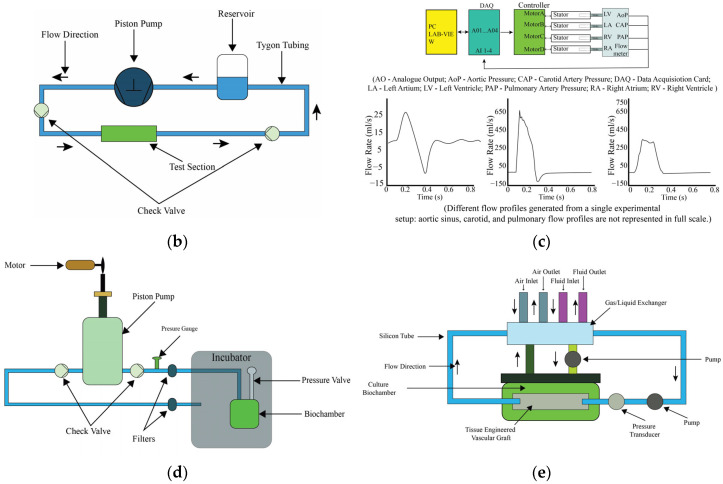
Working principle of piston pump and examples of modifications. (**a**) Generic piston pump configuration, (**b**) schematic of a new pulsatile hydrostatic pressure bioreactor using piston pump [30], (**c**) schematic of control system and generated flow profiles for in vitro cardiovascular emulation [33], (**d**) schematic diagram of pulsatile hydrostatic pressure bioreactor components used for vascular tissue-engineered construction [31], (**e**) schematic of the perfusion-based bioreactor system [32].

Piston pumps work through a reciprocating motion within a cylinder, which facilitates the fluid movement. In the configuration of these pumps, they normally have both an inlet and an outlet valve, and they manage fluid entry and exit during distinct phases of the operational cycle. The fluid moves into the pump body when the inlet valve is open, during the upward movement. Conversely, the inlet valve closes when the piston returns to its original position during the second half of the cycle and the inlet valve closes while the outlet valve opens, enabling fluid movement as illustrated in Figure 5a. From this valve arrangement, unidirectional flow is observed, as documented in references. It is possible to engineer piston pumps with multiple pistons to generate reverse or negative directional flow. By using this phenomenon, application scope and versatility in fluid management systems of piston pumps increases. The amplitude and frequency of the piston pump could be controlled by its operational speed and the dimensions of its stroke, for a characteristic output in the form of sinusoidal wave. This versatility gives control over the flow dynamics, making piston pumps versatile in various applications. Despite their functional advantages, piston pumps are susceptible to contamination risks due to the moving parts inside their design. Considering these aspects requires attention, especially in applications where sterility is critical, such as in bioreactors and biomedical experiments.

Piston pumps have been utilized effectively in the applications of bioreactor systems. Chaudhury [30], Shaikh [31], and Song [32] have utilized piston pumps to generate unidirectional flow in bioreactor setups. Chaudhury et al. [30] developed a programmable, custom physiological wave profile generator based on piston pump technology, designed to facilitate in vitro studies of various heart valve designs. This inventive tool allows for precise simulation of physiological conditions, which enable the researchers to analyze the performance and efficacy of different heart valve designs under controlled laboratory conditions; the system diagram is depicted in Figure 5b.

In a more complex application, Ruiz et al. [33] demonstrated the pump’s capability to mimic cardiac flow patterns in a system incorporating four piston pumps. This setup exemplifies the pump’s ability to generate not only unidirectional but also complex and negative directional flows shown in Figure 5c. Shaikh constructed a novel bioreactor system, as depicted in Figure 5d, employing a range of components including a pressure piston, filters, semi-compliant tubes, a one-way valve, pressure monitors, silicone gasket sealants, and a bio-chamber. This system was designed to cultivate tissue constructs under a controlled pressure environment, aiming to enhance the mechanical characteristics of the cultured tissue. The innovative approach demonstrates the potential of creating a more physiologically relevant environment for tissue growth, thereby improving the functional properties of the engineered tissue constructs. This system exemplifies the integration of mechanical stimulation into tissue culture practices to better mimic in vivo conditions. Song engineered a bioreactor designed for gas–liquid and liquid–liquid exchange, incorporating a system powered by two stepper motor-driven piston pumps. This setup is intended to simulate specific pressure conditions within in vitro experiments, thereby enhancing the applicability of the study’s findings to biological systems. The use of stepper motors allows for precise control over the piston movement, ensuring that the pressure conditions can be accurately mimicked according to the experimental requirements. The schematic diagram of this innovative bioreactor system, illustrating its components and their configuration, is presented in Figure 5e. This design highlights Song’s contribution to advancing bioreactor technology by integrating mechanical elements to replicate physiological conditions more closely. In certain experimental configurations, piston pumps are utilized specifically to induce pulsations within the flow, while other pump types like peristaltic pumps or gear pumps are employed to maintain the mean flow. This tandem operation allows for a broader range of flow control and pattern generation. Additionally, incorporating a reservoir within the piston pump system can facilitate prolonged flow maintenance, enabling periodic fluid alterations. This setup resembles a closed-loop system, further enhancing the pump’s utility in extended experimental setups.

Vignali et al. [34] developed a left ventricular pump system, combined with a 3d printed mock loop, to replicate patient-specific aortic flow for in vitro analysis. This system was engineered to reproduce any custom physiological or patient-specific flow and pressure profiles, and was validated using in vivo MRI data. The custom piston pump system can replicate any adult or pediatric heart condition, which will help to analyze and simulate complex aortic heart conditions. Kizilski et al. [35] developed a system to mimic pediatric right ventricular outflow. They also have used a custom-made piston pump with a 3d printed right ventricular outflow tract for simulating and evaluating pulmonary heart replacement situations. Fanni et al. [36] developed a system to study blood flow in pulmonary arteries of patients with abnormal blood flow conditions or Tetralogy of Fallot. In their setup, they used a combination of in vitro and in silico (computer-simulated) models to simulate how blood flows through the arteries. For the pumping system, they used a custom piston pump to generate patient-specific physiological flows.

### 4.3. Diaphragm Pumps

**Figure 6:**
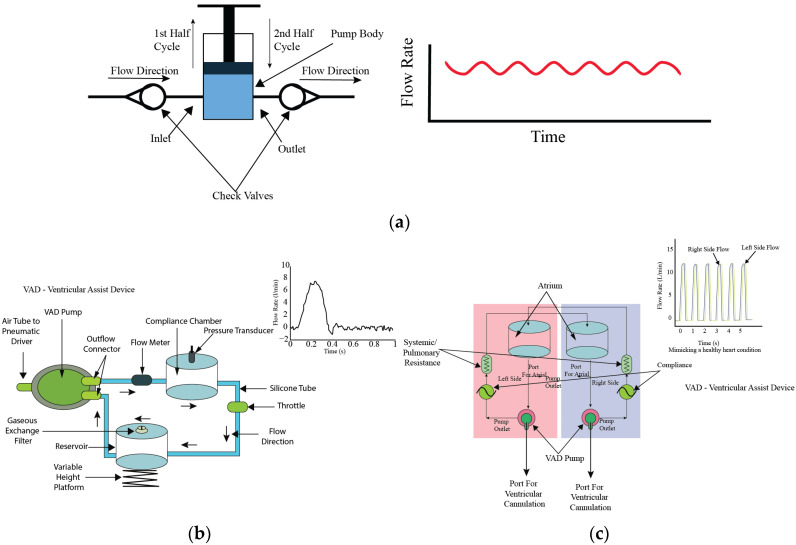
Working principle of diaphragm pump and examples of modifications. (**a**) Generic construction of diaphragm pump, (**b**) schematic of the perfusion-based bioreactor system: shows the components of the integrally designed pulsatile perfusion-based bioreactor system developed for the successful creation of small diameter tissue-engineered vascular vessels [37]; (**c**) schematic of the circulatory mock loop for biventricular device testing: figure presents the schematic of a circulatory mock loop designed for testing biventricular devices under various heart conditions [38].

Diaphragm pumps represent a distinct category of fluid transfer devices, primarily differentiated from piston pumps by their method of operation. While both types of pumps are designed with a single inlet and outlet, and are equipped with valves to facilitate unidirectional flow, diaphragm pumps replace the reciprocating piston with a flexible diaphragm that directly interacts with the fluid. This unique mechanism significantly minimizes the risk of contamination, making diaphragm pumps particularly advantageous for sterile applications, as depicted in Figure 6a. The operation of diaphragm pumps involves the rhythmic movement of a diaphragm, powered by either mechanical, hydraulic, or pneumatic means, to create a variable volume within the pump chamber. As the diaphragm contracts, it increases the chamber volume, generating a vacuum that opens the inlet valve and draws fluid in. Conversely, when the diaphragm expands, it decreases the chamber volume, forcing the fluid out through the outlet valve. This process ensures a steady flow of fluid with generally lower flow capacities compared to piston pumps due to the diaphragm’s limited range of motion. Despite this trade-off in flow capacity, diaphragm pumps are extensively utilized in medical and experimental settings, especially where accurate emulation of cardiac valve conditions is essential. For instance, Kaasi et al. [37] effectively employed a diaphragm pump to replicate heart valve conditions, achieving pulsatile flow rates ranging from 0 to 8 L per minute. This achievement underscores the pump’s relevance in producing physiologically accurate in vitro models for heart valve tissue engineering, as depicted in Figure 6b. Moreover, the inherent lower contamination risk of diaphragm pumps, as compared to other contact pumps, renders them highly suitable for experiments necessitating extended liquid flow durations. This aspect is exemplified by Kado et al. [38], who designed a mock loop system with two ventricular assist devices to simulate the full cardiovascular environment for optimal tissue culture, shown in Figure 6c. Similarly, Ruel et al. [39] presented the use of a diaphragm pump in a system to mimic cardiovascular flow, capable of managing flow rates from 0 to 500 milliliters per minute.

### 4.4. Syringe Pumps

**Figure 7:**
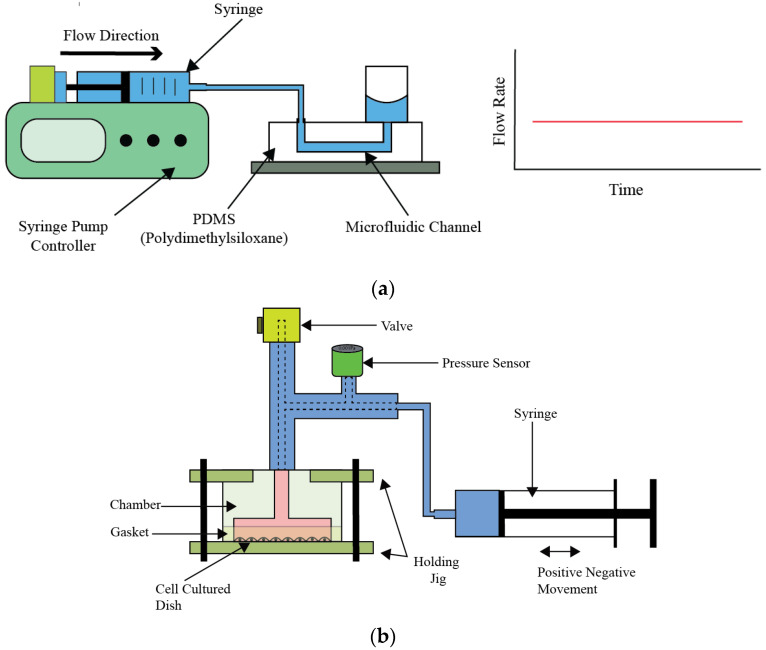
Working principle of syringe pump and examples of modifications. (**a**) Generic construction of syringe pump, (**b**) schematic diagram of the experimental system for applying hydrostatic pressure to cells, showing the experimental setup designed to apply hydrostatic pressure (HP) to endothelial cells, mimicking pressure therapy conditions [41].

Syringe pumps, a subset of contact pumps, can regulate fluid flow with high precision, a characteristic that renders them invaluable across a spectrum of scientific research. These devices excel in open-loop systems, where they are designated to administer the liquid exclusively contained within the syringe, as depicted in Figure 7a. This operational constraint arises from the fundamental design of syringe pumps, which propels liquid by applying positive pressure, thereby facilitating a controlled, linear flow. Although this operational principal bears resemblance to that of piston pumps, a notable distinction is the syringe pump’s inability to utilize self-refilling, confining its operational duration to the syringe’s volume capacity. Manufacturers offer syringe pumps with a diverse array of linear flow capabilities, ranging from extremely low to notably high rates.

The efficacy of syringe pumps in experimental research is well-documented. For instance, Hung et al. [40] utilized a syringe pump in a polydimethylsiloxane (PDMS) cell culture study, assessing the effects of different flow rates on cell proliferation over an eight-day timeframe. Employing flow rates from 0.13 to 0.2 microliters per minute, the experiment successfully generated pulsatile flow patterns, albeit without accurately mimicking cardiovascular dynamics. Similarly, Yoshino et al. [41] leveraged a syringe pump to establish various pressure environments, facilitating the exploration of cell cycle dynamics under distinct conditions, as shown in Figure 7b. Conversely, Baeckert et al. [42] observed a startup delay in the initial operation phase of a syringe pump. Despite this early setback, the device consistently maintained a stable flow rate throughout the experiment, highlighting an important operational consideration for experiments that demand immediate and precise flow adjustment. Expanding on the working principle, syringe pumps operate by mechanically moving the syringe plunger through either a stepper motor or a screw-driven mechanism. This movement exerts a force on the plunger, precisely controlling the rate at which the fluid is expelled. The design allows for meticulous adjustment of flow rates, facilitating the delivery of minute volumes over extended periods or rapid infusion as required.

### 4.5. Vaccum Pumps

**Figure 8:**
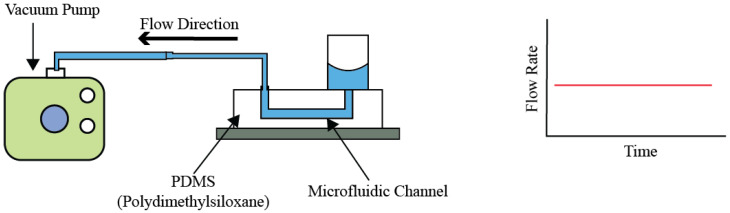
Working Principle and Generic Construction of Vacuum Pump.

Vacuum pumps utilize a unique mechanism to move fluids, fundamentally relying on the creation of negative pressure, or a vacuum, to facilitate flow. This method stands in contrast to the positive pressure mechanisms employed by syringe and piston pumps, where flow is generated through direct force on the fluid, shown in Figure 8. The operational essence of vacuum pumps involves a direct interaction with the fluid alongside the engagement of various mechanical parts, presenting challenges related to potential contamination and mechanical wear over time. These pumps are typically incorporated into open-loop systems, which generally include a liquid reservoir, the targeted system for fluid movement, and the vacuum pump. The operation principle hinges on the liquid maintained within the reservoir, ensuring that with periodic replenishment, the system can support sustained fluid flow. Despite their operational advantages, vacuum pumps face limitations, attributed to their size and heightened contamination risks.

The versatility of vacuum pumps in research environments is evidenced by several experimental applications. For instance, Huh et al. [43] effectively employed a vacuum pump in an air–liquid mixing experimental setup. This arrangement succeeded in generating a concentrated stream of liquid at high velocity, enriched with particles and cells, over a four-day period, achieving flow rates between 6 and 20 milliliters per hour. Further illustrating the adaptability of vacuum pumps, King et al. [44] devised a system incorporating multiple vacuum pumps to emulate pulsatile-like flow conditions. This innovative approach facilitated the examination of cellular behaviors under dynamic fluidic environments, underscoring the capability of vacuum pumps to meet the demands of complex experimental designs. Operating on the principle of negative pressure, vacuum pumps draw fluid into a system by reducing the pressure within the pump chamber, causing the external atmospheric pressure to push the fluid into the chamber. This mechanism is critical for applications requiring the removal of air or other gasses from a sealed volume to create a vacuum or for transporting fluids in systems where direct contact with a mechanical component is less desirable.

### 4.6. Gear Pumps

Gear pumps, a subset of contact pumps, are characterized by their direct mechanical engagement with the liquid, a trait that inherently increases the risk of contamination. This interaction, along with the potential for gear wear over time, necessitates stringent sterilization protocols to mitigate contamination risks between uses. One of the key advantages of gear pumps is their capability to produce bidirectional flow, achieved by simply reversing the gears’ rotation direction. This versatility is invaluable in diverse experimental configurations, providing a solution for systems requiring variable flow directionality. Despite contamination concerns, gear pumps excel in delivering high flow rates, with capabilities extending to liters per minute. Additionally, they can produce both constant and pulsatile flows, functioning effectively alone or in conjunction with other pumps like syringe or piston pumps to enhance flow dynamics.

**Figure 9:**
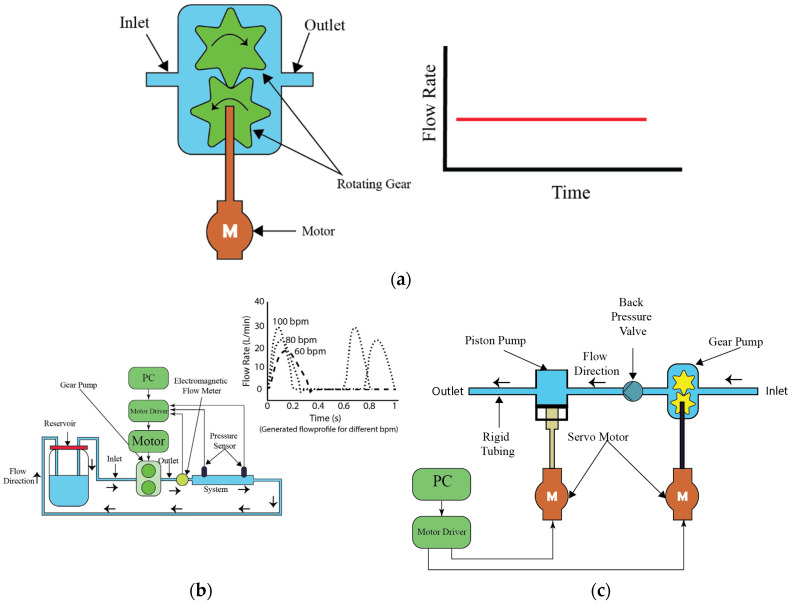
Working principle of gear pump and examples of modifications. (**a**) Generic construction of gear pump, (**b**) schematic diagram of the experimental setup for pulse duplication system, presenting the schematic diagram of the experimental setup for an affordable pulse duplication system designed for in vitro cardiovascular experiments [46], (**c**) schematic diagram of the flow pumping system for physiological waveforms, showing the schematic diagram of the flow pumping system designed to generate physiological waveforms using conjugated operation of gear pump and piston pump [45].

In scientific investigations, gear pumps have been instrumental. Tsai et al. [45], for instance, integrated a gear pump to establish a foundational flow within a system engineered for pulsatile flow patterns, complemented by a piston pump that introduced pulsatility, achieving flow rates up to 15 milliliters per minute, as depicted in Figure 9a. This setup’s modularity allowed for flow rate customization through component selection. Choi et al. [46] showcased a gear pump operating solo to create pulsatile flow, demonstrating the pump’s impressive flow rate range from 0 to 30 L per minute, highlighting its superior flow capacity relative to other pumps, as shown in Figure 9b. Gear pumps operate by meshing gears to move fluid through the pump. This process involves the gears coming together at the inlet side, creating a vacuum that draws fluid into the pump. As the gears rotate, they carry fluid around the outer periphery of the gear casing to the outlet side, where the meshing gears force the fluid out. This mechanism allows for efficient fluid transfer at high volumes, making gear pumps an essential tool in applications requiring robust flow management.

The comparison of characteristics of contact pumps, highlighting the flow rate, flow patterns, shear stress, application sectors, and key advantages and disadvantages, is shown in Table 2.

## 5. Emerging Pump Systems

The landscape of pump technology is experiencing significant positive changes with the introduction of novel pumping mechanisms, including both contact and non-contact categories. Using these advanced pumps, we can deliver pulsatile or constant flows.

**Figure 10:**
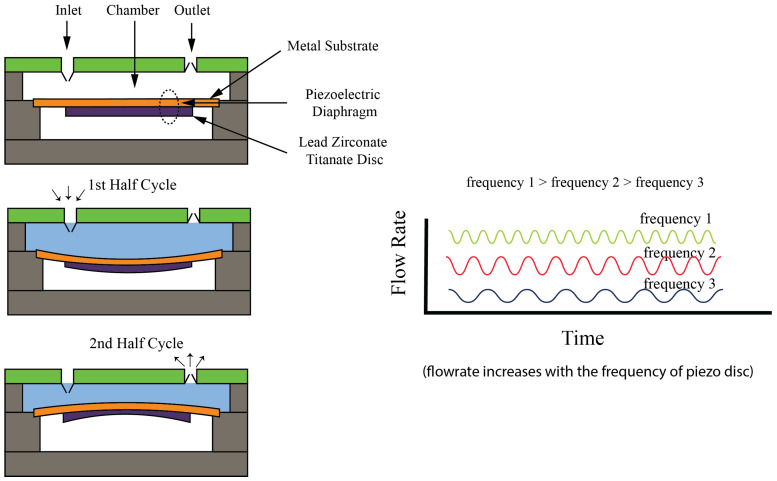
Working Principle and Generic Construction and Operation of Piezo Pump [52].

The piezoelectric pump, one of the remarkable pumps within the non-contact pump category, generates positive pressure to move liquid through it, which includes both an inlet and an outlet so that it can generate a unidirectional flow. This type of pump has the quality of minimal mechanical movement, which, while generating lower flow rates, also makes the use of high operating voltages compulsory, frequently reaching into the kilovolt range. However, its capability to offer precise flow control and diminish unwanted pulsations by adjusting voltage and frequency stands out as a significant advantage. The pump’s compactness and precise flow management make it especially suitable for microfluidic applications that require exact fluid mixing. The working principle of the piezoelectric pump utilizes the function of piezoelectric effect, where, due to an electric field applied across a piezoelectric material, a mechanical deformation is caused in the material. By this deformation, pressure is generated within the pump, which causes the liquid to move. Specifically, the piezoelectric element, which is mostly a ceramic or crystal, expands or contracts in response to an applied voltage, creating a pressure differential that propels the fluid from the inlet to the outlet, illustrated in Figure 10. The precise control of the voltages makes it easy to control the pump’s output, enabling precise adjustments to the flow rate. This unique operation mechanism does not require direct contact between the pump’s moving parts and the fluid, which minimizes the risk of contamination, further enhancing the piezoelectric pump’s appeal in applications where sterility and precision are important. Kassis et al. [52] demonstrated the pump’s adaptability by coupling it with a Raspberry Pi for control, achieving a flow range from 0 to 4000 microliters per minute. This piezoelectric pump is ideal for extended battery-powered operations due to its low energy consumption and open-loop configuration. A low-frequency-driven piezoelectric pump was developed by Chen et al. [53], having a flexible valve (LDPPFV), for applications in microfluidics and drug delivery. The pump was able to achieve a maximum flow rate of 18.1 mL/min, having precise control ability with high particle tolerance. However, there were issues such as reverse leakage, which required attention to ensure reliable operation. The focus of the design of pump was on improving flow efficiency while maintaining compactness, to make it efficient for portable biomedical devices. Wang et al. [54] introduced a resonant piezoelectric diaphragm pump capable of transferring liquid with a maximum flow rate of 186.8 mL/min. The resonant frequency operation was utilized by the pump to enhance output pressure, which makes it ideal for microfluidic and gas transfer applications. There were advantages in system integration due to its compact design, although the complexity of the structure and noise during operation were still challenges. The versatility and precision of piezoelectric pumps are highlighted in the study of both pumps but emphasize the need for further optimization in flow stability and noise reduction.

**Figure 11:**
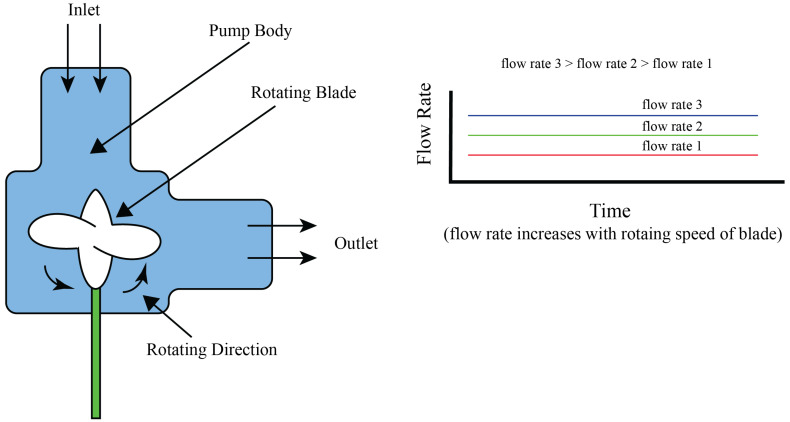
Working Principle and Generic Construction of I-Cor Diagonal Pump [55].

I-Cor diagonal pumps have shown usefulness in biomedical applications as well. The operational principle of the I-Cor diagonal pump involves a unique diagonal flow path that distinguishes it from traditional centrifugal or axial flow pumps [Figure 11]. This design incorporates a rotor positioned diagonally within the pump’s housing, creating a helical flow pattern as it rotates. When activated, the rotor’s movement generates centrifugal forces that propel the blood or fluid diagonally across the pump from the inlet to the outlet. This diagonal flow path is engineered to reduce shear stress on blood cells, a critical consideration in minimizing hemolysis and other potential damage to the blood during pumping. Additionally, the I-Cor diagonal pump’s design allows for synchronization with the patient’s cardiac cycle, enabling it to augment or support the heart’s pumping action in a way that mimics natural physiological conditions closely. This feature is particularly beneficial for patients requiring ventricular assist devices, as it can adapt to the varying demands of the cardiovascular system, providing support that is more in tune with the body’s natural rhythms. Utilized primarily for ECG-synchronized pulsatile flow, Force et al. [55] have demonstrated its application as a temporary cardiac assist device. While its adoption is mainly within medical contexts, constrained by relatively high costs and operational difficulties, the pump has a distinct advantage of being capable of delivering flow rates ranging from 0 to 2500 milliliters per minute. This range enables it to simulate various arterial flow conditions, making it invaluable in cardiovascular research and patient care. Cremers et al. [56] tested a novel I-Cor system utilizing a diagonal pulsatile pump for cardiogenic shock and ECMO applications. The pump, synchronized with the heart’s ECG signal, was shown to significantly increase coronary artery blood flow compared to non-pulsatile modes, especially during ventricular fibrillation. The system provided consistent circulatory support and diastolic augmentation, suggesting potential benefits for patients in cardiac arrest. These findings demonstrate the pump’s ability to improve perfusion, though further human trials are needed to confirm clinical outcomes.

**Figure 12:**
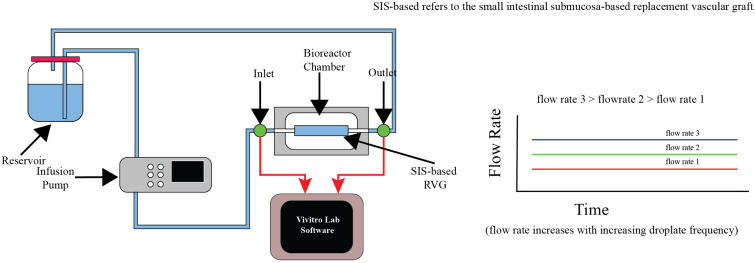
Working principle and schematic of the fluidic device for SIS-based vascular graft evaluation, presenting the schematic of the fluidic device and the necessary accessories for cell seeding on the SIS-based vascular graft [57].

In another innovative application, Riveros et al. [57] developed and characterized a fluidic device aimed at evaluating the biological properties and hydrodynamics of SIS-based vascular grafts. The system incorporated an infusion pump and an agitation mechanism to simulate physiological conditions, promoting HUVEC cell growth on the grafts. The device achieved a pulsatile flow rate of 1200 mL/h and generated wall shear stress conditions resembling those found in native vessels. In silico CFD validation confirmed the experimental results, with the device providing a cost-effective platform for vascular graft testing. Despite its advantages, the system requires precise calibration to maintain consistent flow dynamics across experiments [Figure 12].

These examples show the dynamic evolution of pump technologies, where novel designs and their applications are continually being explored. Although these pumps, from the precision-oriented piezoelectric pump to the medically focused I-Cor diagonal pump and the adaptable infusion pump, are still in the early stages of broader application, they have high transformative potentials in fluid dynamics for biomedical applications. Table 3 presents a comparison of various characteristics of emerging pump types, highlighting their flow rates, flow patterns, operational advantages, disadvantages, and specific application areas across different sectors.

## 6. Conclusions

In vitro pump systems play an important role in replicating physiological cardiovascular flows and both non-contact and contact pumps have their own specific advantages and limitations. Non-contact pumps, including peristaltic and centrifugal pumps, provide the benefit of reduced contamination risk by isolating the fluid from mechanical parts, but have the limitation of lower flow rates and have limited flexibility in adjusting flow characteristics. Contact pumps, including syringe and gear pumps, are appreciated in applications which require exact fluid handling, such as tissue culture and medical interventions, due to their capability of precisely controlling flow rates. Limitations are observed when the risk of contamination is considered, due to the direct interaction of pumps with the fluid.

Emerging pump technologies, like piezoelectric and I-Cor diagonal pumps, overcome many limitations of the traditional systems. Piezoelectric pumps, with their ability to control very low flow rates with high accuracy without any direct contact with the fluid, are good for microfluidic and biomedical applications where precise and contamination-free handling of minute fluid volumes is crucial; as opposed to traditional contact pumps, they offer superior sterility and minimal mechanical complexity. On the other hand, I-Cor diagonal pumps, designed specifically for medical applications, are superior to both contact and non-contact pumps in replicating physiological conditions, particularly in ventricular assist devices. Their capability of synchronizing with the cardiac cycle enables more natural, pulsatile flow patterns, offering significant advantages in medical and cardiovascular research environments. These pumps overcome the limitations of traditional contact pumps, and have precision, sterility, versatility, and adaptability.

## Figures and Tables

**Table 1:** Comparison of characteristics of non-contact pumps.

Reference	Pump Type	Flow Rate	Flow Pattern and Duration	Generated Shear Stress Magnitude	Used Sectors	Advantages	Disadvantages
Bouhrira [2]	Peristaltic pump with linear actuator	1–6 mL/min	Pulsatile, physiological, 18 h	Variable shear stress (CFD data)	Blood–brain barrier, endothelial studies	Low-cost, mimics physiological waveform	Limited to low flow rates
Helms [4]	Pulsatile peristaltic pump	64 mL/min	Pulsatile, 10 days	-	Vascular tissue engineering	Promotes smooth muscle differentiation	Requires custom bioreactor setup
Eoh [5]	Peristaltic pump	0.74 mL/min	Pulsatile flow, 30 days	3–5.5 × 10^−4^ dyne/cm^2^	Vascular tissue engineering	Enhanced elastin and ECM production	Requires bioreactor and scaffold setup
Gu Wei [6]	Braille-based peristaltic pump	74 nL/min	Pulsatile, continuous, 3 weeks	-	Microfluidic cell culture	Portable, programmable, long-term culture	Limited scalability, channel constriction
Chong [7]	Reciprocating roller pump	0.88–4.25 L/min	Pulsatile, physiological, continuous	-	Cardiopulmonary bypass, ECMO	Reproduces realistic blood flow waveforms	Limited to pre-programmed flow profiles
Balagaddé [8]	Peristaltic Pump	250 µm/s	Continuous, semi-continuous circulation, over 200 h	-	Metabolic culture studies	Prevents biofilm formation, long-term monitoring	Requires precise control of microfluidics
Gómez-Sjöberg [9]	On-chip peristaltic pump	6.5 nL/s	Pressure-driven, peristaltic, 7–9 days	0.05 N/m^2^	Stem cell research, differentiation studies	Fully automated, precise media exchange	Bubble formation, slower for flushing tasks
Ju [10]	Surface tension-driven micropump	45 µm/s	Continuous, slow flow	-	Electrofusion, plant cells	Simple, cost-effective, no peripherals required	Limited to slow-flow applications
Ju [11]	Surface tension-driven micropump	45 µm/s	Continuous, reversible	-	Microfluidics, cell transport	Simple, passive, low power requirement	Prone to backward flow at low volume ratios
Park [12]	Osmotic pump	0.15 µL/h	Continuous, laminar, 10 days	Low, minimized	Stem cell differentiation	Long-term stable gradient, minimal handling	Complex initial setup
Park [13]	Osmotic pump	0.15 µL/h	Continuous, gradient, 8–21 days	-	Stem cell differentiation	Stable long-term gradient, minimal media use	Requires careful pressure balance
Rhee [14]	Centrifugal force-based system	-	Hydrodynamic, centrifugal, 30–300 s	-	Cell positioning, chemotaxis	Simple setup, adaptable for multiple cell types	Cell viability can be affected at high RCF
Ren [15]	Centrifugal microfluidic pump	-	Vortical, spiral toroidal, up to 24 h	-	Cell culture, mixing studies	Short lag phase, efficient mixing	Requires optimized flow patterns
Song [16]	Braille-based peristaltic pump	4.9 µL/s	Pulsatile, peristaltic, 48–72 h	1–12 dyne/cm^2^	Endothelial cell culture	High control over shear stress, multi-loop setup	Limited to shear stress up to 12 dyne/cm^2^
Law [17]	Computer-controlled roller pump	-	Pulsatile, physiological, continuous operation	-	Doppler ultrasound studies	Simulates physiological flow, easily cleaned	Limited reverse flow capability
Hoenicka [18]	Peristaltic pump	40–60 mL/min	Continuous, pulsatile, 4–8 days	0.05–0.22 Pa	Blood vessel metabolism	Maintains endothelial cell viability	Requires precise viscosity control
Wang [19]	Peristaltic Pump	30 µL/min	Continuous, gradient flow, up to 24 h	0.54–6 dyne/cm^2^	Cell culture, force transduction	Real-time observation, spatial shear gradients	Limited scalability
Farcas [20]	Low-pulsatility peristaltic pump	119.5 mL/min	Steady, laminar flow, 8–24 h	20 dynes/cm^2^	Endothelial cell culture	Accurate cell elongation, realistic artery model	Introduces minor pressure fluctuations
Ota [21]	Peristaltic pump	1.1 mL/min	Micro-rotational flow, over 1 day	-	Spheroid formation, cell culture	Precise control of spheroid size, long-term culture	Clogging in channels, requires shredder channels
Futai [22]	Braille-based pump	50 nL/min	Pulsatile, 8–21 days	-	Portable cell culture	Portable, long-term culture, minimal maintenance	Limited scalability
Heo [23]	Braille-based peristaltic pump	500 nL/min	Recirculating, continuous, 10 h	-	Endothelial cell culture	Prevents osmolality shifts, sub-microliter recirculation	Requires parylene coating to prevent evaporation
Mehta [24]	Braille-based peristaltic pump	220 µL/s	Continuous, gradient-driven, up to 12 h	-	Microfluidic bioreactors, cell culture	Precise oxygen control, small volume setup	Requires extensive calibration and control
Sasaki [25]	Miniaturized infusion pump	30 µL/h	Intermittent flowOver 102 h	0.17 dyne/cm^2^	Cell culture, endothelial studies	Small, portable, easy-to-use system	Limited scalability, risk of air bubble formation
Unger [26]	Peristaltic elastomeric pump	2.35 nL/s	Pulsatile, peristaltic	-	Microfluidics, cell culture	Low dead volume, fast actuation, durable	Requires pneumatic control system
Wu [27]	Pneumatic peristaltic micro-pump	185.1 μL/h	Pulsatile, peristaltic, up to 48 h	-	3D cell culture, micro bioreactors	High-throughput, uniform medium distribution	Complexity in scaling up, time delay due to fluid resistance

**Table 2:** Comparison characteristics of contact pumps.

Reference	Pump Type	Flow Rate	Flow Pattern and Duration	Generated Shear Stress Magnitude	Used Sectors	Advantages	Disadvantages
Wolf [28]	Micro-centrifugal pump	10-2000 mL/min	Pulsatile, physiological, up to 25 h	-	Tissue-engineered vascular graft conditioning	Compact, transportable, disposable	Requires batteries for autonomous operation
Diamantouros [29]	Pulse-free centrifugal pump with magnetic actuator	50–1000 mL/min	Pulsatile, supra-physiological, up to 4 weeks	0.067–13.5 dyne/cm^2^	Vascular graft durability testing	Realistic pressure waveforms, compliance monitoring	Complex setup, requires multiple sensors
Chaudhury [30]	Piston-based pulsatile pump	850 mL/s	Pulsatile, physiological, continuous operation	-	Aortic flow experiments	High Reynolds number, MRI-compatible	Requires large-scale setup, limited to high flow rates
Shaikh [31]	Pulsatile hydrostatic pressure pump	-	Pulsatile, cyclic, 24–48 h	-	Vascular tissue engineering	Compact, easy sterilization, flexible setup	Limited to pressure-based conditioning only
Song [32]	Pulsatile perfusion-based pump	10 mL per cycle	Pulsatile, physiological, 2 weeks	-	Vascular tissue engineering	Precise control, high patency	Complex setup, requires motor-driven control
Ruiz [33]	Linear motor-driven pump	5 L/min	Pulsatile, physiological	-	Cardiovascular simulation	Simulates normal and pathological conditions	Complex system setup required
Vignali [34]	Custom-made piston pump	5 L/min	Pulsatile, physiological, continuous operation	-	Patient-specific hemodynamics	High versatility generating patient-specific flow	Complex setup, high maintenance
Kizilski [35]	Custom-made piston pump	-	Pulsatile, physiological, continuous operation		Pediatric right ventricular outflow tract (RVOT) simulation	Accurate simulation of pediatric heart conditions	Complex setup, requires precise calibration
Fanni [36]	Custom-made piston pump	-	Pulsatile, patient-specific	-	Pulmonary artery simulations	High accuracy in replicating patient-specific flows	Complex setup and calibration required
Kaasi [37]	Ventricular assist device (VAD)	-	Pulsatile, physiological, 4 days	-	Heart valve tissue engineering	Mimics ventricular pressures, dynamic conditioning	Complex setup, potential contamination risks
Kado [38]	Pneumatic pumps (Abiomed AB5000)	4.2 L/min	Pulsatile, physiological	-	Mechanical circulatory device testing	Customizable for different heart failure conditions	Requires extensive calibration and monitoring
Ruel and Lachance [39]	Custom diaphragm pump (Windkessel model-based)	160 mL/cycle	Pulsatile, physiological		Tissue-engineered heart valve development	Accurate physiological flow and pressure waveforms	Requires complex setup and calibration
Hung [40]	Syringe pump	0.2 mL/min	Continuous, uniform flow, 7.5 days	-	High-throughput cell culture	Cost-effective, uniform microenvironment	Requires careful flow rate calibration
Yoshino [41]	Hydrostatic pressure system	-	Constant pressure, cyclic, 24 h	-	Endothelial cell research	Controlled positive and negative pressure	Requires careful sealing to maintain pressure
Baeckert [42]	Syringe infusion pump	1 mL/h	Continuous, low flow, variable duration	-	Pediatric critical care	Precise for small-volume infusions	Prone to start-up delays and flow irregularities
Huh [43]	Air–liquid two-phase flow pump (vacuum pump)	-	Pulsatile, air–liquid, continuous operation	-	Flow cytometry, microfluidics	Disposable, low-cost, minimal contamination risk	Limited to hydrophobic channel systems
King [44]	Microfluidic flow-encoded switching (vacuum pump)	-	Continuous, laminar, long-term culture	<0.1 dyne/cm^2^	Dynamic cell culture	High-throughput, scalable, parallel flow control	Requires precise pressure control
Tsai and Savaş [45]	Gear-piston pump system	300 mL/s	Pulsatile, physiological, continuous operation	-	Vascular flow experiments	Accurate waveform replication, easy tuning	Sensitive to flow loop impedance
Choi [46]	Gear pump with feedback control	40.9 L/min	Pulsatile, physiological	-	Cardiovascular simulations	Affordable, precise replication of waveforms	Unable to replicate reverse flow
Chodzyński [47]	Custom piston pump with linear motor	40–700 mL/min	Pulsatile, physiological, continuous operation	-	Cardiovascular simulation	Accurate reproduction of coronary blood flow	Requires complex real-time control system
Drost [48]	Arduino-controlled gear pump	1.5 L/min	Pulsatile, physiological, continuous operation	-	Vascular access hemodynamics	Affordable, customizable	Requires semi-automatic system identification
Lee [49]	Moving-actuator type pump	8 L/min	Pulsatile, physiological, continuous operation	-	Ventricular assist device (VAD), TAH	Single device for LVAD (Left Ventricular Assist Device), RVAD (Right Ventricular Assist Device), BVAD (Biventricular Assist Device), or TAH (Total Artificial Heart)	Requires complex compliance and control systems
Mechoor [50]	Real-time programmable pulsatile pump	500 mL/min	Pulsatile, physiological	-	Cardiovascular experimentation	Accurate, adjustable flow patterns	Requires feedback control for waveform accuracy
Kurniawan [51]	Syringe Pump	300 µL/min	Continuous, programmable. Continuous operation	-	Microfluidics, drug delivery	High precision, customizable flow rates	Limited to small-scale application

**Table 3:** Comparison of emerging pumps characteristics.

Reference	Pump Type	Flow Rate	Flow Pattern and Duration	Generated Shear Stress Magnitude	Use Sectors	Advantages	Disadvantages
Kassis [52]	Piezoelectric pump (PiFlow system)	1–3000 mL/min	Pulsatile, programmable, continuous operation	-	Microfluidics, cell culture	Low-cost, scalable, biocompatible	Limited to moderate flow resistance systems
Chen [53]	Low-frequency-driven piezoelectric pump with flexible valve	18.1 mL/min	Pulsatile, low frequency, continuous operation	-	Microfluidics, drug delivery	Precise flow control, high particle tolerance	Reverse leakage needs control
Wang [54]	Resonant piezoelectric diaphragm pump	186.8 mL/min	Pulsatile, resonant, continuous operation	-	Gas transfer, microfluidics	High output pressure, compact design	Complex structure, working noise
Force [55]	ECG-synchronized i-cor diagonal pump	200–1800 mL/min	Pulsatile, physiological, continuous operation	-	Pediatric cardiac assist	Higher hemodynamic energy, reduces afterload	FDA (Food and Drug Administration) approval pending, off-label use
Cremers [56]	Diagonal pulsatile pump (i-cor system)	0–8 L/min	Pulsatile, ECG-synchronized, continuous operation	-	Cardiogenic shock, ECMO (Extracorporeal Membrane Oxygenation)	Increased coronary perfusion, precise control	Requires synchronization with ECG (Electrocardiogram), complex setup
Riveros [57]	Baxter infusion pump	1200 mL/h	Pulsatile, physiological, continuous operation	12 dynes/cm^2^	Vascular graft testing	Cost-effective, easy assembly	Requires calibration for flow consistency
